# Chronotype Differences and Symptom Network Dynamics of Post-Pandemic Sleep in Adolescents and Young Adults

**DOI:** 10.3390/jcm13175020

**Published:** 2024-08-25

**Authors:** Maxime Windal, Aurore Roland, Marise Laeremans, Giovanni Briganti, Charles Kornreich, Olivier Mairesse

**Affiliations:** 1 Faculty of Psychology, Université Libre de Bruxelles, 1050 Brussels, Belgium; 2 Brain, Body and Cognition, Department of Psychology, Faculty of Psychology and Educational Sciences, Vrije Universiteit Brussel, 1050 Brussels, Belgium; aurore.roland@vub.be (A.R.); olivier.mairesse@vub.be (O.M.); 3 Brussels University Consultation Center, Department of Psychology, Faculty of Psychology and Educational Sciences, Vrije Universiteit Brussel, 1050 Brussels, Belgium; 4 Independent Researcher, 2980 Zoersel, Belgium; marise.laeremans@hotmail.com; 5 Chair of Artificial Intelligence and Digital Medicine, Faculté de Médecine, Université de Mons, 7000 Mons, Belgium; giovanni.briganti@ulb.be; 6 Laboratoire de Psychologie Médicale et Addictologie, Faculté de Médecine, Université Libre de Bruxelles, 1050 Brussels, Belgium; charles.kornreich@chu-brugmann.be; 7 Department of Clinical Sciences, Faculté de Médecine, Université de Liège, 4000 Liège, Belgium; 8 Laboratoire de Psychologie Médicale et Addictologie, Centre Hospitalier Universitaire Brugmann, 1020 Brussels, Belgium; 9 Vital Signs and PERformance Monitoring (VIPER), LIFE Department, Royal Military Academy, 1000 Brussels, Belgium

**Keywords:** COVID-19, adolescent, sleep, insomnia, chronotype, lockdown, mental health

## Abstract

**Background:** Social restrictions during the COVID-19 pandemic resulted in altered sleep patterns and mental health challenges, particularly among adolescents and young adults. Our objective was to examine the potential difference in insomnia prevalence and sleep patterns in this population between the first COVID-19 lockdown and the post-lockdown period, with a focus on chronotype. Additionally, we explored the network of sleep-related differences between these two periods. **Methods:** A total of 946 respondents participated in our online questionnaire. We performed mixed ANOVA, Ising network and Directed Acyclic Graph (DAG) analyses. **Results:** Respondents reported going to bed earlier, waking up earlier, sleeping less, and feeling less mentally tired than during the lockdown. The severity of insomnia symptoms did not change. The lethargic chronotype reported more insomnia symptoms, depressive feelings, and agitation than others. Mental fatigue was the central symptom in the Ising network and served as the parent node in the DAG. **Conclusions:** Post-lockdown, adolescents and young adults have shifted to earlier sleep and wake times with reduced overall sleep, and they experience fewer depressive feelings and less agitation, though insomnia symptoms remain unchanged. Participants who reported increased irritability or poorer sleep quality during confinement also reported similar or diminished attentional capacities compared to their usual levels.

## 1. Introduction

To prevent the propagation of the COVID-19 virus, governments took measures like restricting social life, teleworking, and following classes online from home. This aspect of the pandemic has led to mental health complaints and sleep problems across all ages [1,2,3,4]. Sleep habits also changed: in general, individuals went to bed and woke up later. In a previous study in our group, we found that young adults had the greatest delay of all adult age groups in bed- and rise times during the first lockdown (in spring 2020), also reporting sleeping more during than before the lockdown [5]. It is hypothesized that the absence of in-person schooling or the flexibility to attend classes remotely provided adolescents and young adults with an opportunity to adopt sleep schedules more closely aligned with their innate biological rhythms [5,6,7,8].

Many processes in the human body follow a rhythm of approximately 24 h, known as the circadian rhythm. Both internal (endogenous) and external (exogenous) factors synchronize this rhythm to a 24 h day. However, individuals exhibit differences in their internal time or phase of entrainment, known as chronotypes [9]. Predominantly recognized chronotypes include morning and evening types (M-types and E-types), as well as individuals falling in between (intermediate types or I-types). However, [10] introduced additional classifications: daytime type (also referred to as afternoon types or A-types), daytime sleepy type (napper types or N-types), highly active type (vigilant types or V-types), and moderately active type (lethargic types or L-types). The distribution of these chronotypes is not uniform across age groups, with a peak in eveningness observed during late adolescence/early adulthood [11]. We posit that this peak may explain why, given the opportunity during lockdown, adolescents and young adults chose the latest bed- and wake times compared to other age groups, allowing them to get sufficient sleep [5].

Ultimately, government-imposed measures were lifted, and normalcy largely resumed. Consequently, our research aimed to explore post-lockdown sleep patterns and mental health, concentrated on adolescents and young adults, given the significant alterations observed in their sleep patterns and mental health during the lockdown period. Although other studies have explored the post-lockdown sleep patterns of our target demographic before us, we posit their data collection might not have coincided with the period when schools returned to normal operations [12,13]. As mentioned earlier, it is probably the closing of schools and transition to remote classes that allowed adolescents and young adults to change their sleep patterns. The Organization for Economic Co-operation and Development (OECD) [14] praised Belgium for having managed to keep schools open for much longer than other nations. Yet, even in Belgium, it is only in 2022 that secondary schools and universities fully resumed normal operations. Therefore, it seems to us premature to investigate post-lockdown sleep patterns in 2020 and in 2021 and more accurate to conduct such studies in 2022, when schools and universities had fully reopened. A study [15] investigated sleep patterns in the general population in spring 2022, but did not specifically focus on our target demographic and only measured the three most known chronotypes (M-, E- and I-types). Finally, our collected data will focus on the current perception that our respondents have of their sleep, whether it concerns sleep habits or difficulties, during the past remarkable societal conditions such as lockdown and social isolation.

In this study, we investigate (1) how the prevalence of clinical insomnia and sleep patterns evolved in adolescents and young adults between the initial COVID-19 lockdown (spring 2020) and the subsequent post-lockdown period (spring 2022), and (2) how chronotype influences sleep pattern changes across these periods. Finally, (3) we investigated the network of sleep-related differences between the two periods using an undirected (Ising) and directed (directed acyclic graph) network to investigate the interconnectedness of symptoms, their conditional and directed dependencies.

## 2. Materials and Methods

### 2.1. The Participants

The sample in this study consists of Dutch-speaking adolescents between 14 and 24 years of age. There were no exclusion criteria. Out of a total of 948 respondents that started the survey, 498 participants completed it (52.5%). Among them, there were 334 women (67.2%), 153 men (30.8%), 8 participants who preferred not to disclose this information (1.6%), and 2 feel neither male nor female (0.4%). The average age of the participants was 16 years (SD = 2.81). A distribution of chronotype according to age can be found in Table 1.

### 2.2. Materials and Procedure

This study was approved by the ethics committee of the UVC Brugmann Hospital (Brussels, Belgium) and is in accordance with the Declaration of Helsinki. A 52-item survey, available in both Dutch and French, was launched on 23 February 2022, disseminated through social media channels (Facebook and Instagram) and via email communication with secondary schools. The deadline for inclusion of completed questionnaires in our analysis was set for 21 March 2022. Notably, despite the availability of a French version, only four French-speaking individuals commenced the survey, and their lack of completed responses necessitated their exclusion from the analysis.

After requiring biographical information, subsequent queries focused on sleep-related parameters including bedtime (BT), wake time (WT), sleep onset latency (SOL), total sleep time (TST), time in bed (TIB), and the level of depressive feelings and agitation during the initial lockdown in spring 2020. Additionally, participants were presented with the seven-item Insomnia Severity Index (ISI), a 5-point Likert scale ranging from 0 (no problem) to 4 (very severe problem), evaluating the severity of difficulties with sleep initiation, maintenance, early morning awakening, overall satisfaction with sleep, impact of sleep disturbances on daily functioning, the perceptibility of sleep issues by others, and the distress engendered by such difficulties [16]. Participants were then asked about any alterations in sleep patterns since the onset of the lockdown. Those affirming changes were further questioned with dichotomous items to ascertain the nature of these alterations. The questionnaire repeated inquiries on BT, WT, SOL, TST, TIB, depressive feelings, and agitation, alongside the ISI items, this time contextualized to the post-lockdown period of spring 2022. The survey concluded with the Single Item Chronotyping test (SIC [10]). Non-essential items for the current analysis were omitted from this report. Participation in the study was voluntary and free, with respondents retaining the option to withdraw at any time.

### 2.3. Statistical Analysis

A mixed-design ANOVA was performed using SPSS version 28 to examine the influence of chronotype on potential changes in sleep pattern between peri- and post-lockdown. Symptom networks comprising the 12 questions related to the perception of changes in sleep patterns between the lockdown and the present were estimated with R (Version 4.1.3). The 12 variables in this network are binary, denoted as 0 for ‘no change’ and 1 for ‘change’.

### 2.4. Ising Network

The Ising model is a network estimated from binary data that are commonly 0 and +1 in the field of research in psychology. The edges between variables in an Ising model are estimated from logistic regressions with domain {0, 1}. R-packages *bootnet* (version 1.5) and *Q-graph* (Version 1.9.3) were used to estimate and plot the ISING model [17,18], following the methodology outlined by [19]. The index of expected influence was used to determine the central symptom by using the *networktools* package (version 1.5) in R. This index is calculated by summing the weights of the edges connected to a specified node, by taking into account the sign of the edge weight (unlike the strength index which takes the absolute value of edge weight). The R package *bootnet* (version 1.5) was used to evaluate the robustness and the stability of the network. To achieve this, a non-parametric bootstrapping technique was used to evaluate the accuracy of the edges within the model. The technique involves creating a new data set based on 95% confidence intervals to randomly resample observations on these data. This procedure thus allows for visualizing which (quantity of) edges significantly differ from those estimated by resampling, indicating a lack of robustness. Next, a Stability Coefficient of Correlation (CS-C) is obtained using the case-dropping method, which assesses the stability of the network. This coefficient indicates the highest proportion of the sample that could be removed without significantly affecting the original correlations of the different indices (expected influence in our case), thus maintaining a correlation equal to or greater than 0.7 with a 95% probability. On this subject, [17] recommend that this CS coefficient should ideally be above 0.5, and should not be lower than 0.25. Finally, bootstrapped difference tests (*n* = 1000) were conducted to measure the properties of the networks such as the weight of the edges and the expected influence index [17].

### 2.5. Directed Acyclic Graphs

Directed Acyclic Graphs (DAGs) are networks which allow the representation of a directed and potentially causal structure of the probabilistic relationships between variables [20]. The description of the DAGs will be done through the two approaches used in our two previous studies: the first describes the importance of the relationships between two nodes within the model. This importance will be quantified using the Bayesian Information Criterion (BIC) score. A BIC score will thus be calculated for each of the arcs. This score is calculated relative to the entire model from which this arc would have been removed, and thus represents a relative measure of the fit to this model. In other words, the higher the score, the more important this arc is for the structure of the model. The second approach measures the directional probability with which an arc points from node A to node B. This probability is therefore a percentage and is obtained by calculating the frequency of occurrences where this arc points in this specific direction, among all the networks estimated by bootstrapping. The DAGs are estimated using algorithms, and for this study, we chose to use the Bayesian Hill-Climbing algorithm, in accordance with [21] and prior psychological research [22,23,24,25]. In this way, we utilized the R package bnlearn (version 4.8.1) [26,27]. This package employs the bootstrap function that estimates the structural features of the model by adding, removing, and reversing edges, to optimize the final model based on the BIC score. To ensure the stability of the resulting DAG, we will perform a bootstrap of 10,000 iterations (with replacement, to avoid local optima) in accordance with [21]. Then, these 10,000 estimated networks will be compiled to construct the final network structure using a two-step procedure. To begin, we will quantify the frequency with which an arc connecting A and B (regardless of its direction) appears among the 10,000 bootstrapped networks (which corresponds to the “strength” variable in R). Only frequencies exceeding the optimal cutoff (favoring high sensitivity and high specificity) calculated by the approach of [28] will be retained for the final network. Now that the most important connections are retained, their respective directional probabilities will be calculated and displayed. As a result, only directional probabilities greater than or equal to 51% will be displayed in the final network. Finally, to ease the interpretation of the DAG, we opted for the same method as used in our previous study [29]: firstly, the thickness of the arcs in the network is proportional to the BIC value (the thicker the arc, the more it indicates a negative BIC value), and secondly, we added the values of the directional probabilities to each of the arcs on the same graph.

## 3. Results

### 3.1. Mixed ANOVA

**Figure 1:**
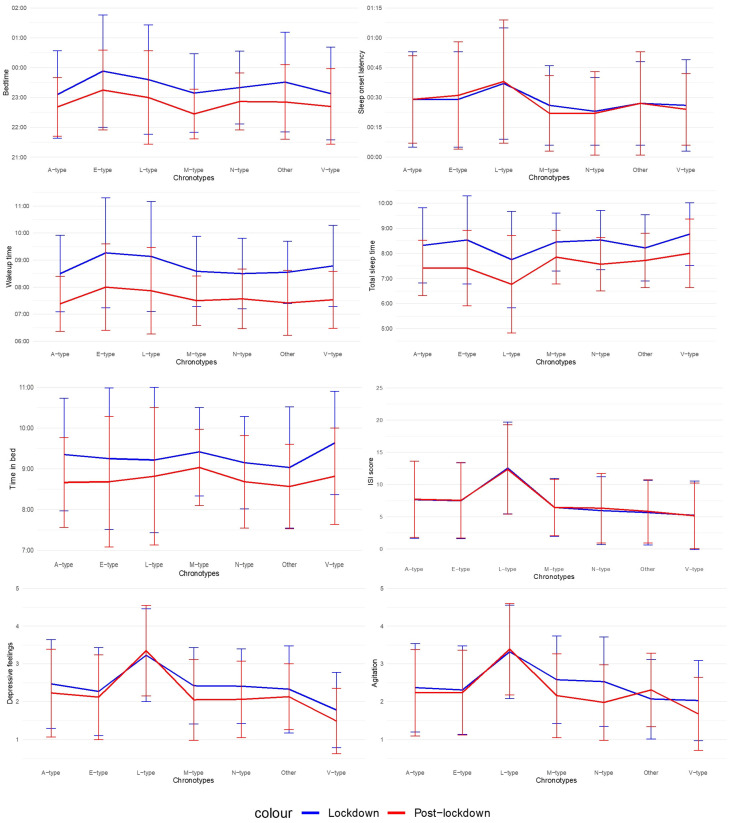
Mixed ANOVA results. Note. M-types = Morning types, E-types = Evening types, A-types = Daytime or Afternoon types, N-types = Daytime Sleepy or Napper types, V-types = Highly Active or Vigilant types, L-types = Moderately Active or Lethargic types.

Results of the mixed ANOVAs are presented in Table 2 and in Figure 1. Post-lockdown, adolescents and young adults report going earlier to bed, waking up earlier, sleeping less, spending less time in bed, feeling less depressed and less agitated. No significant difference between the total mean score for ISI during the lockdown (M = 7.43, SD = 5.97), and during post-lockdown (M = 7.48, SD = 5.88) was observed. There was also no significant difference in the severity of insomnia symptoms, manifested by a 13.9% current prevalence of insomnia (moderate to severe complaints) for our entire sample and an insomnia prevalence of 13.7% during the lockdown in the same group. Except for TIB, there was a main effect of chronotype for all variables. For depressive feelings and agitation, this effect was large. Post-hoc analyses with Bonferroni corrections reveal that Evening types (E-types) report a later BT than Daytime or Afternoon types (A-types) (*p* = 0.001), Morning types (M-types) (*p* = 0.01) and Highly Active or Vigilant types (V-types) (*p* = 0.02) and a later WT than A-types (*p* = 0.001). Moderately Active or Lethargic types (L-types) report needing on average 14 min longer to fall asleep than Daytime Sleepy or Napper types (N-types) (*p* = 0.004). Furthermore, they report sleeping less than all the other types, so unsurprisingly, their ISI scores are higher than those of all the other types. They also report feeling more depressed and agitated than all the other types. In contrast, V-types report feeling less depressed than all the other types. Agitation is the only variable exhibiting an interaction effect: individuals who did not fit in any of the six chronotypes (t(44) = −1.45, *p* = 0.15), the L- (t(56) = −0.41, *p* = 0.68), E- (t(107) = 0.71, *p* = 0.48) and A-types (t(114) = 1.22, *p* = 0.23) did not report a difference in agitation. On the other hand, the N- (t(65) = 3.53, *p* < 0.001), V- (t(62) = 2.53, *p* = 0.01) and M-types (t(42) = 2.73, *p* = 0.01) report a decrease in agitation.

For the sake of completeness, we also performed a mixed ANOVA with lockdown as within-subjects factor and age (14–15 y vs. 16+ y) as between-subjects factor. However, the interaction effects, if present, were negligible.

### 3.2. Network Analysis

Respondents (*n* = 414) who endorsed the question “Has your sleep pattern changed between the lockdown and usual times?” and subsequently completed the questions regarding these changes were included in the estimation of the following networks.

#### 3.2.1. Ising

**Figure 2:**
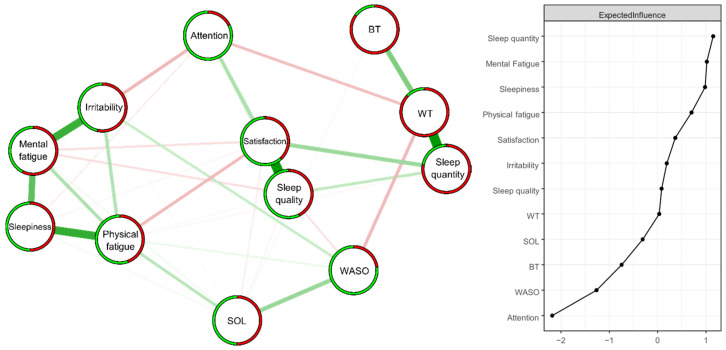
Estimated ISING network (*n* = 414) structure of 12 items regarding changes in sleep patterns between the lockdown and usual conditions (**left** panel) and the corresponding Expected Influence indices (**right** panel) as standardized z-scores. Positive and negative associative relationships were colored in green and red, respectively. The colored ring around each node represents the probability of obtaining 0 (in green = “no”) or 1 (in red = “yes”).

The model consists of 12 variables and 36 connections (out of a maximum of 66 non-zero edges, which is the theoretical maximum of possible connections) (Figure 2). Among these 36 connections, 20 are positive and 16 are negative. The ring divided into two parts around the nodes visually represents the proportion of respondents who answered no = 0 (in green) or yes = 1 (in red). The probabilities of endorsing each item, as well as their exact wordings, are in Table 3. A bootstrap (nboots = 1000) was performed for Edge and Expected Influence. These bootstrap analyses attest to the stability of Edge (CS-C = 0.67) and Expected Influence (CS-C = 0.52) [17] (see Supplementary Materials).

The strongest positive edges (superior to two standard deviations) were “Sleep quality”—“Sleep satisfaction” (2.43) and “Sleep quantity”—“Waking time” (2.42), followed by “Physical fatigue”—“Sleepiness” (1.86), Mental fatigue”—“Irritability” (1.79), and “Mental fatigue”—“Sleepiness” (1.36) (superior to one standard deviation). The strongest negative edges (superior to one standard deviation) were “Irritability”—“Attention” (−1.27), “Waking time”—“WASO” (−1.26), “Physical fatigue”—“Sleep satisfaction” (−1.23) and “Waking time”—“Attention” (−1.19).

The nodes with the highest Expected Influence values (superior to one standard deviation) were “Sleep quantity” (1.15) and “Mental Fatigue” (1.02) (Figure 2).

#### 3.2.2. DAGs

**Figure 3:**
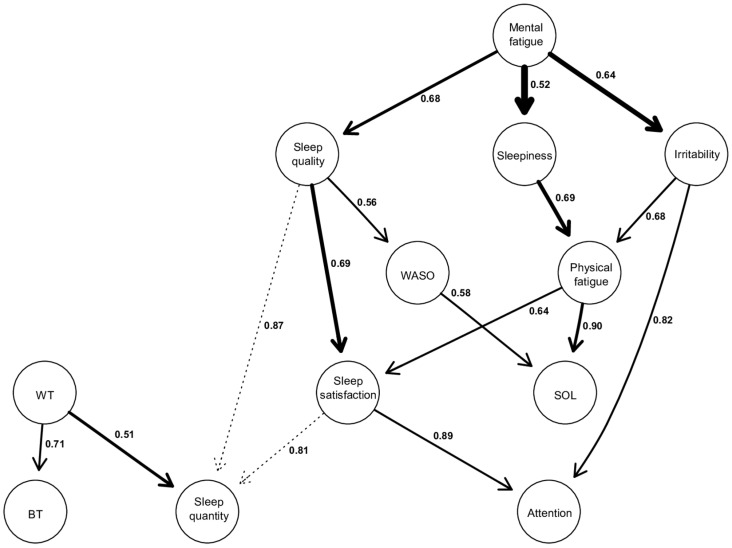
Directed acyclic graphs (DAGs). The thickness of an arrow is proportional to the BIC score, thus reflecting its importance to the overall structure of the network. Non-significant arcs, whose strength is slightly below the calculated threshold, are drawn in dotted lines. The values associated with each edge correspond to directional probabilities, representing the proportion of an arrow pointing in that direction across all 10,000 iterated networks in which this relationship (regardless of the arrow’s orientation) was observed.

The model consists of the same 12 variables as the ISING model (Figure 3). The edges present in this graph were retained because their strength was higher than the threshold (0.5) calculated from the approach of Scutari and Nagarajan (2013). This means that the relationships displayed are present in at least 50% of the 10,000 iterations (which represents a minimum of 5000 occurrences). The complete set of indices characterizing each edge (strength, directional probability, and BIC score) can be found in the Supplementary Materials.

Firstly, the thickness of the arrow is proportional to the BIC value corresponding to this arrow. The thicker the arc, the more negative the BIC score, and the more it contributes to the overall network structure [25]. In this way, the most important arrows in the network connect “Mental Fatigue” to “Sleepiness” (BIC = −69.20), “Mental Fatigue” to “Irritability” (BIC = −67.62), “Sleep quality” to “Sleep satisfaction” (BIC = −61.63), and “Sleepiness” to “Physical Fatigue” (BIC = −49.70).

Secondly, the values on the arrows correspond to directional probabilities. For example, the most directed arrow connects “Physical fatigue” to “SOL” with a directional probability of 0.90, which means that “Physical fatigue” points to “SOL” in 90% of all occurrences where this relationship (“Physical fatigue”—“SOL”) was observed during the 10.000 iterations. The other strongly directed edges include “Satisfaction of sleep” to “Attention” (0.89), “Sleep quality” to “Sleep quantity” (0.87), “Irritability” to “Attention” (0.82). However, there are also relationships with a low directional probability (close to 0.5), indicating more of a bidirectional link rather than a unidirectional relationship. This is exemplified by “Waking time” to “Sleep quantity” (0.51), “Mental fatigue” to “Sleepiness” (0.52), “Sleep quality” to “WASO” (0.56).

Thirdly, the activation of a node within a DAG is conditionally dependent on the activation of the nodes located above it. Indeed, each node activation in a DAG has a probability that is conditionally dependent on those corresponding to its parent node [25,30]. Thus, from a structural point of view, we can describe the following results: “Mental fatigue” appears to be the parent node of the model. It can propagate through three other nodes, namely “Sleep quality”, “Sleepiness”, and “Irritability”.

The relationship between “Mental fatigue” and its child nodes is significant for the network structure, given their high BIC scores, especially the relationships between “Mental fatigue” and “Sleepiness” and “Mental fatigue” and “Irritability” (BIC = −69.2, and BIC = −67.62, respectively). These latter two relationships are the most important within the entire network. Furthermore, the directional probability of “Mental fatigue” and “Sleep quality”, as well as “Mental fatigue” and “Irritability”, is 0.68 and 0.64, respectively. However, the one between “Mental fatigue” and “Sleepiness” is close to 0.52, indicating a relatively strong bidirectional dependence with a BIC score of −69.2.

Also, it is observed that there are several child nodes in the global network, such as “BT”, “Sleep quantity”, and “Attention”. This means that these nodes are solely dependent, and no other nodes are dependent on them. Regarding “BT”, there is only one parent node, which is “WT”, with a directional probability of 0.71.

As for “Attention”, it is dependent on the parent node of mental fatigue and can be activated through two possible pathways, both of which lead to participants reporting that their attention is either equivalent or decreased during confinement. The first pathway starts with “Mental fatigue”, which, when perceived as increased during confinement, propagates through perceived “Sleep quality” as equivalent or decreased (0.68), leading to a similar effect on “Sleep satisfaction” (0.69), and ultimately resulting in attentional capacities perceived as equivalent or decreased during confinement (0.89). The other pathway also starts with perceived increased “Mental fatigue”, which propagates towards increased “Irritability” (0.64), subsequently leading to attentional capacities perceived again by respondents as similar or decreased during confinement (0.82).

Finally, it is also observed that “SOL” is another child node in the model but positioned higher in the hierarchy compared to “Attention”, “BT”, or “Sleep quantity”. This superior position in the hierarchy indicates that “SOL” potentially has other nodes to which it could connect below it, but these hypothetical connections are not displayed as they are below our calculated threshold score. “SOL” can be activated through two different pathways, both stemming from the parent node of “Mental fatigue”. However, these two pathways lead to opposite effects on “SOL”. The first pathway involves perceived “Mental fatigue” as equivalent or decreased during confinement, which propagates through an increase in “Sleep quality” (0.68), which in turn leads to “WASO” considered stable or decreased (0.56), resulting in “SOL”, which is perceived by our respondents as equivalent or decreased during confinement (0.58). The other pathway starts with an increase in “Mental fatigue”, propagating through an increase in “Sleepiness” (0.52), subsequently increasing “Physical fatigue” (0.69), and ultimately resulting in an increase in “SOL” (0.90).

## 4. Discussion

Our respondents currently report earlier sleep schedules, characterized by earlier bedtimes and wake-up times compared to the lockdown period. Respondents also report sleeping less, spending less time in bed, and feeling less depressed and less agitated. Chronotype had an effect on each of our variables (except TIB). Agitation is the only variable showing an interaction effect: the N-, V-, and M-types exhibit a decrease in agitation, in contrast to other chronotypes. The L-Type shows higher values across all variables (except TIB) compared to other chronotypes. Furthermore, from a network perspective, the variables with the greatest influence are “Sleep quality” and “Mental fatigue”. The directed network (DAG) consists of the same variables as the first undirected network (Ising) and is characterized by “Mental fatigue” as the parent node and “Attention” as the child node of the model. Moreover, the most robust relationships link “Mental fatigue” to “Sleepiness”, “Mental fatigue” to “Irritability”, and “Sleep quality” to “Sleep satisfaction”. Finally, a perceived change in attentional capacities during the lockdown may be dependent, through two different symptom cascades, on an increase in mental fatigue.

### 4.1. Sleep Habits after the Pandemic

In spring 2020, both young adults [5,8] and adolescents [1,8,31] reported going to bed and waking up later than before the pandemic. Adolescents and young adults now report an earlier BT and WT than during that lockdown. While we cannot confirm whether they have fully reverted to their pre-lockdown BT and WT as we did not inquire about those times, they probably did. We hypothesized that since schools and universities were closed and classes had to be attended from home, this age group, which tends to have a later chronotype [11]—a finding corroborated by our study—was able to adopt a sleep schedule that aligns better with their chronotype. However, with the resumption of in-person classes, they are now compelled to adhere to earlier bedtime and wake-up schedules.

In our previous study [5], we found a higher TIB and TST among young adults during the lockdown than compared to before. This increased subjective TST during the lockdown aligns with findings from other studies on young adults [8] and adolescents [8,31]. Interestingly, this was accompanied by a decrease in sleepiness in adolescents [8,31], but not in young adults [8]. In our current study, we observe a decrease in both TIB and TST. While in this study we did not collect data on pre-lockdown TIB and TST in this particular cohort, we hypothesize that these values have returned to their pre-lockdown levels.

Throughout the lockdown period, a surge in insomnia-related complaints was documented among young adults [5,8]. Conversely, findings for adolescents were mixed, with two studies reporting no significant variation [1,8] and another study identifying an increase [31]. In the post-lockdown period, sleep onset latency and insomnia symptoms remain the same as during lockdown. Notably, only the L-type exhibits scores of 15 or higher on the ISI, indicating clinically significant insomnia as measured by the ISI. According to the third edition of the International Classification of Sleep Disorders [32], among adolescents and young adults, an SOL of more than 20 min is considered to be a sleep-initiating difficulty. Given that all chronotypes for both time periods have a mean SOL of more than 20 min, sleep-initiating difficulties appear to be a prevalent problem both during and after the lockdown in this age group.

With the exception of the L-types, our sample did not report high levels of depression and agitation during or after the lockdown. Nevertheless, they reported a slight improvement in these mental health variables.

### 4.2. Influence of Chronotype

Most respondents in our study are E- or A-types, aligning with the higher prevalence of a later chronotype in this age group reported in other studies [11]. Surprisingly, we did not find a peak in eveningness in late adolescence/early adulthood, but rather among 14–15-year-olds. Among 21–24-year-olds, the most prevalent chronotype was the N-type. These differing results may be attributed to the fact that respondents were presented with a broader range of chronotype options beyond the commonly studied M- and E-types. Additionally, we observed a slightly higher percentage of respondents not choosing any of the six chronotypes compared to the 5% reported in the study by [33].

As expected, E-types reported later or, at least, the same BT and WT as the other types. Notably, L-types stood out with the longest SOL, lowest TST, highest total ISI score and the highest reported levels of depressive feelings and agitation. This chronotype is characterized by having low or moderate energy throughout the entire day [33]. Our results suggest that individuals with this chronotype may experience daytime tiredness, associated with insomnia, depression and anxiety symptomatology. Therefore, choosing this diurnal preference in the questionnaire might be more the reflection of poor mental or sleep-related health than the result of genetic factors [34] or developmental processes [11].

There was no interaction effect between chronotype and lockdown, except for agitation. This contrasts with the results of [15] who, in the general population, found only an interaction effect for TST. Our results indicate that for all variables, aside from agitation, all chronotypes reacted in a similar manner to the lifting of the lockdowns. Interestingly, even morning types, who typically prefer early BT and WT, report advancing their BT and WT post-lockdown. This suggests a probable delay in their BT and WT during the lockdowns when they were no longer required to be physically present at school. School start times might thus even be too early for morning adolescents.

### 4.3. Network Analysis

Both the directed and undirected networks yielded similar results. Firstly, more than half of the respondents who reported perceiving a change between lockdown and their usual routine experienced increased mental fatigue during confinement. Secondly, this perceived change is significantly associated with other perceived changes within our two networks. It is, on the one hand, one of the two variables most connected to our entire ISING network and, on the other hand, serves as the parent node in our Directed Acyclic Graph (DAG), implying that the variables it points to are conditionally dependent on its state.

“Attention” appears as a child node of “Mental fatigue”. This can be interpreted as the presence of changes in attentional capacities (i.e., the “child” node) implying more strongly the presence of changes in mental fatigue (i.e., the “parent” node) than vice versa. Or, in other words, individuals reporting changes in attentional capacities are more inclined to report mental fatigue rather than the other way around.

The Ising network informs us about the alleged direction of these dependencies, revealing that starting from mental fatigue, two different pathways with distinct changes lead respondents to perceive their attentional capacities as either unchanged or impaired during confinement. Both pathways are primarily dependent on mental fatigue perceived as increased during confinement compared to usual. This increase in mental fatigue either propagates towards sleep perceived as of lower quality (or at least considered equivalent) or towards an increase in irritability. In both cases, these changes result in attentional capacities judged, mostly by our respondents, as poorer or equivalent during confinement compared to their usual routine.

Fatigue, which should not be confused with sleepiness, is a common complaint in the general population [35]. It is described as a condition in which maintaining a motor or mental effort level gets more difficult in acute and very energy-demanding tasks [36,37]. The increase in mental fatigue during lockdown among our population is not surprising given the new, stressful, and isolating context imposed by lockdowns and the necessary sanitary and social distancing measures to adhere to. Young adults, who place a high importance on social connections and are more actively involved in social activities compared to other age groups [38,39], were significantly impacted by the lockdown and home confinement during the pandemic [40]. This situation could lead to a diminished sense of connection with their peers and friends, heightened social isolation, and feelings of emotional loneliness. Consequently, these factors may contribute to a heightened risk of fatigue and various mental health issues [41,42].

Our model also indicates that mental fatigue could have had a negative impact on sleep quality, and that sleep perceived as of lower quality could affect attentional functions. These associations are not surprising, as one of the major symptoms of insomnia is precisely the idea that sleep problems have repercussions on daily life activities, such as mood and cognitive functions [43,44]. Regarding the association between mental fatigue and sleep quality, a study was conducted on a population of Brazilian women during the COVID-19 pandemic, which observed both high levels of fatigue and an association of this fatigue with various depressive and anxious symptoms, as well as lower sleep quality [45].

Our model also indicates that perceiving one’s attentional capacities as impacted, or equivalent, is conditionally associated with the presence of increased irritability. The presence of increased irritability, in turn, more likely implies an increase in mental fatigue during confinement, rather than the other way around. Moreover, a study investigated the subjective impact that confinement may have had on cognitive functions and highlighted a subjective alteration of executive functions, including attention. These attention complaints were also associated with depressive and anxious symptoms, of which irritability is a part [46].

Our results also suggest that sleep onset latency (SOL) could be dependent on perceived mental fatigue during confinement, with two different pathways leading to opposite effects on SOL. In the first pathway, perceiving an extended Sleep Onset Latency (SOL) is more likely to result in increased physical fatigue. Furthermore, the presence of this increased physical fatigue is dependent on the presence of heightened daytime sleepiness. Finally, experiencing more daytime sleepiness is more inclined to be associated with experiencing increased mental fatigue during confinement. This result may seem paradoxical but could be explained by the fact that increased mental fatigue experienced during confinement encourages a sedentary lifestyle, such as that observed during lockdowns. This sequence of circumstances conditions individuals to expend less energy, which could impact physical fitness, including cardiovascular condition and muscle strength, potentially making daily activities more tiring [47]. From this perspective, it is not surprising to observe that SOL is increased given the extensive literature on the known beneficial effects of physical activity on sleep, particularly sleep onset latency [48,49].

In the second pathway, however, perceiving mental fatigue as equivalent or even lower during confinement had a rather beneficial effect. In this situation, perceiving one’s sleep latency as shorter or equivalent during confinement suggests a greater likelihood of reduced awakenings throughout the night. Additionally, experiencing fewer or an equal number of awakenings after falling asleep is conditionally dependent on perceiving one’s sleep quality as better. Lastly, the presence of better sleep quality implies a greater likelihood of perceiving less, or equivalent, mental fatigue during the confinement period rather than the other way around. These results are not surprising and highlight that adolescents who experienced less or equivalent mental fatigue during confinement were the same individuals who subsequently had better sleep quality, fewer awakenings, and required less time to fall asleep. Not perceiving an increase in mental fatigue during lockdown most likely had a protective effect on sleep, especially sleep onset latency.

### 4.4. Strengths and Limitations

We measured insomnia using a standardized, reliable and validated questionnaire, the ISI [50]. This study is, to our knowledge, the first attempt to investigate post-lockdown sleep. However, only approximately half of our sample completed the questionnaire. Nevertheless, with 496 participants aged 14 to 24, the sample size remains relatively substantial. We used three different types of statistical analyses. This variety allows for a deeper understanding of the complex relationships between sleep patterns, mental health and chronotypes.

Our research utilized a cross-sectional method. Due to the absence of longitudinal data, establishing cause-and-effect relationships was not feasible, despite the use of DAGs. Furthermore, there might be a bias in participants’ responses concerning the lockdown period, as this information was collected retrospectively. The relevance of the study’s findings may be limited, specifically to Dutch-speaking adolescents and young adults in Belgium, and only during a certain phase of the COVID-19 pandemic. No questions were asked in our questionnaire about the use of sleep medication, which considering the slight increase of its use in this population is a limitation [51].

In our network analyses, a positive response from our respondents indicates that they perceived a change between the current period and the lockdown, specifically indicating a change in the direction asked in the question. For example, we did not ask whether their level of mental fatigue had changed, but whether it had increased. Consequently, a negative response to these questions does not necessarily imply that this variable decreased during confinement. In fact, the variable may also be equivalent between the two periods. A negative response to our question thus might signify that there was no change or that the change happened in the opposite direction of our question.

The strengths of our study make it a valuable contribution to the understanding of how major societal disruptions like pandemics can affect key aspects of adolescent and young adult life. However, the limitations should be considered when interpreting the results.

### 4.5. Implications and Suggestions for Further Research

The misalignment of school start times with adolescents’ biological rhythm forces them to wake up too early, resulting in insufficient sleep [52]. Since schools were closed during the lockdown, TST could increase and sleepiness decrease [8,31]. However, with adolescents now required to return to in-person schooling, their TST has decreased once again. This concern affects all chronotypes, including M-types who also had a later BT and WT during the lockdown. Numerous studies already suggest several benefits of later school start times [53]. Our results indicate that these later school start times might be preferred by all chronotypes, even morning types. We thus propose continuing the investigation of the benefits of this schedule change using high-quality research methods [53]. Furthermore, our results suggest that in the chronotypes proposed by [33], choosing the L-type might be the reflection of poor mental and sleep-related health.

Our findings, which highlight an alteration in reported mental fatigue and changes in attentional capacities and sleep during and after lockdown, could be further understood through recent neurobiological theories. The Active Inference Theory, for example, suggests that the brain continuously tests and adjusts its internal models based on external stimuli [54]. In this context, the lockdown environment may have induced dopaminergic neuroplastic changes as an adaptive response to this situation. According to this framework, mental fatigue and attentional impairments might not reflect specific cognitive impairment but could also represent the brain’s active efforts to maintain functional balance amidst these environmental changes. Thus, one might speculate that mental fatigue observed in our study could be interpreted as part of the brain’s attempt to reorganize its resources and preserve functional integrity during a prolonged period of reduced social interaction and increased isolation [55]. Future research focusing specifically on neurophysiological markers and these cognitive and emotional functions could be investigated through network analysis to highlight potential relationships and directionalities.

Finally, and regarding the long-term effects of the pandemic, it would be interesting to look at the role of the socioeconomic status and digital screen usage on these effects, as well as conducting longitudinal research on this topic. This would prevent a potential memory bias.

## 5. Conclusions

Now that the lockdowns are over, adolescents and young adults report going earlier to bed, waking up earlier, sleeping less, spending less time in bed, feeling less depressed and less agitated. The presence of insomnia symptoms did not change. Although the values depended on the chronotype, there was only an interaction effect between chronotype and lockdown for agitation. L-types reported more insomnia symptoms, depressive feelings, and agitation than individuals with other chronotypes. In addition, our respondents experienced more mental fatigue during confinement. This increased mental fatigue is propagated via an increase in irritability or a quality of sleep that was either comparable to or lower than that experienced during the usual period for our respondents. Those who reported having been more irritable or having had a poorer sleep quality during confinement, also reported equivalent or even impaired attentional capacities compared with usual.

## Figures and Tables

**Table 1:** Demographics of participants and chronotypes.

Demographics and Chronotypes	14–15 y (n, %)	16–17 y (n, %)	18–20 y (n, %)	21–24 y (n, %)	Total (N, %)
E-type	49 (25.0)	29 (23.4)	14 (18.2)	16 (16.2)	108 (21.8)
N-type	15 (7.7)	14 (11.3)	12 (15.6)	24 (27.3)	65 (13.1)
A-type	55 (28.1)	28 (22.6)	14 (18.2)	18 (18.2)	115 (23.2)
V-type	34 (17.3)	11 (8.9)	11 (14.3)	7 (7.1)	63 (12.7)
L-type	21 (10.7)	19 (15.3)	10 (13.0)	7 (7.1)	57 (11.5)
M-type	8 (4.1)	10 (8.1)	8 (10.4)	17 (17.2)	43 (8.7)
Other	14 (7.1)	13 (10.5	8 (10.4)	10 (10.1)	45 (9.1)
Total	196	124	77	99	496

Note. y = years, M-types = Morning types, E-types = Evening types, A-types = Daytime or Afternoon types, N-types = Daytime Sleepy or Napper types, V-types = Highly Active or Vigilant types, L-types = Moderately Active or Lethargic types.

**Table 2:** Mixed ANOVA results. Bold is required to highlight significant results (with a *p* value < 0.05); BT = bedtime, SOL = sleep onset latency, WT = wake time, TST = total sleep time, TIB = time in bed, ISI = Insomnia Severity Index.

	Lockdown			Chronotypes			Interaction		
	F(df)	*p*	η2p	F(df)	*p*	η2p	F(df)	*p*	η2p
BT	74.87 (1, 485)	**<0.001**	0.13	3.91 (6, 485)	**<0.001**	0.05	0.58 (6, 485)	0.75	0.01
SOL	0.19 (1, 476)	0.67	< 0.001	3.08 (6, 476)	**0.01**	0.04	0.60 (6, 476)	0.73	0.01
WT	271.04 (1, 491)	**<0.001**	0.36	4.03 (6, 491)	**<0.001**	0.05	0.50 (6, 491)	0.81	0.01
TST	123.38 (1, 491)	**<0.001**	0.20	5.01 (6, 491)	**<0.001**	0.06	1.19 (6, 491)	0.31	0.01
TIB	64.16 (1, 485)	**<0.001**	0.12	0.93 (6, 485)	0.48	0.01	0.82 (6, 485)	0.56	0.01
ISI	0.81 (1, 491)	0.37	0.002	10.64 (6, 491)	**<0.001**	0.12	1.14 (6, 491)	0.34	0.01
Depressive feelings	17.83 (1, 490)	**<0.001**	0.04	15.49 (6, 490)	**<0.001**	0.16	1.48 (6, 490)	0.18	0.02
Agitation	10.02 (1, 490)	**0.002**	0.02	13.29 (6, 490)	**<0.001**	0.14	3.44 (6, 490)	**0.002**	0.04

**Table 3:** The statement of each item presented to the 414 respondents who initially stated that they had perceived a change between the usual period and the lockdown. The probability corresponds to the average of responses (0 and 1), indicating the proportion of these respondents who answered “yes” for this item. “WT” = Waking Time, “BT” = Bedtime, “SOL” = Sleep Onset Latency, “WASO” = Wake After Sleep Onset.

Item	Label	Probability
1. During the lockdown, I got up later than usual.	WT	0.87
2. During the lockdown, I went to bed later than usual.	BT	0.85
3. During the lockdown, I slept longer than usual.	Sleep quantity	0.78
4. During the lockdown, I was more mentally tired than usual.	Mental fatigue	0.57
5. During the lockdown, I was more irritable than usual.	Irritability	0.53
6. During the lockdown, it took me longer to fall asleep than usual.	SOL	0.50
7. During the lockdown, I was sleepier than usual.	Sleepiness	0.50
8. During the lockdown, I was more physically tired than usual.	Physical fatigue	0.44
9. During the lockdown, I slept better than usual.	Sleep quality	0.43
10. During the lockdown, I was more satisfied with my sleep than usual.	Satisfaction	0.38
11. During the lockdown, I woke up more often at night than usual.	WASO	0.23
12. During the lockdown, I was more attentive than usual.	Attention	0.18

## Data Availability

The data is available at the Open Science Framework: https://osf.io/tv2dw (accessed on 13 August 2024).

## Supplementary Materials

The following supporting information can be downloaded at: https://osf.io/tv2dw (accessed on 13 August 2024), strength, BIC and directional parameters of the DAG; correlation stability coefficients.
